# Editorial: Bioactive soft materials and application

**DOI:** 10.3389/fbioe.2023.1248995

**Published:** 2023-07-04

**Authors:** Lihua Li, Thomas Groth, Mingyan Zhao

**Affiliations:** ^1^ Department of Materials Science and Engineering, Jinan University, Guangzhou, China; ^2^ Department Biomedical Materials, Institute of Pharmacy, Martin Luther University Halle-Wittenberg, Halle, Germany; ^3^ Stem Cell Research and Cellular Therapy Center, Affiliated Hospital of Guangdong Medical University, Zhanjiang, China

**Keywords:** hydrogel, nanoparticles, drug delivery, extracellular vesicles, biomedical application, freeze-drying platform

Soft biomaterials represent hydrogels but also a variety of thicker surface coatings based on grafting of hydrophilic macromolecules or their physical assembly by methods like layer-by-layer technique. Soft biomaterials have several advantages that allow uptake and controlled release of bioactive protein-based molecules like growth factors and other kind of drugs. Moreover, soft biomaterials of hydrophilic character allow control of protein adsorption and cell adhesion and interaction which not only can avoid blood coagulation but also tailor immune response or engrafting of the construct to guide tissue regeneration. Another advantage of soft biomaterials is the ability of materials scientists to provide them with stimuli responsive properties that they can react to changes in temperature, pH value, ionic strength and other physical or chemical stimuli to liquefy, control release of drugs or cell interaction depending on the environment. Hence, the aim of the Research Topic “*Bioactive Soft Materials and Application*” is to present the current state of research and development in the field of bioactive soft materials with a perspective focus on material design and their biomedical application.

The Research Topic includes several studies on stimuli responsive hydrogels being used as drug delivery platform for biomedical applications. An interesting work from Li et al. shows the synthesis and formulation of temperature- and pH-responsive chitosan hydrogels that are loaded with doxorubicin and liposome-encapsulated curcumin which demonstrates their potential to be applied for the treatment of solid tumors. Another paper from Zhang et al. described the use of thermosensitive Poloxamer 407 hydrogels for the loading of concentrated growth factors (CGFs) which showed a sustained release of growth factors and demonstrated to be good candidates for the repair of segmental bone defects. In addition, a thermo-responsive and injectable hyaluronic acid/nHAp (HA/nHAp) composite hydrogel was developed by Liu et al. with the incorporation of notoginsenoside R1 (NGR1) used because of its anti-inflammatory properties to reduce TNF alpha production and thus promote bone regeneration. Moreover, Wang et al. reported the fabrication of soft nanoparticles using konjac glucomannan that are loaded with curcumin which can accomplish colonic localization release and target local inflammatory macrophages, showing their potential to be used as oral delivery vehicles for the treatment of inflammatory bowel disease.

An interesting work is about the design of platform for batch fabrication of native tissue mimicking scaffolds. Lin et al. developed a unique freeze-drying system for the fabrication of Haversian system using chitosan/type I collagen composite materials to mimic native bone tissue to enhance bone regeneration. This universal preparation platform based on the directional freezing technology is also expected to be applied for the fabrication of scaffolds with cavities roughly arranged at right angles to each other. In addition, Luo et al. reviewed the latest progress in studying the roles of culture-condition stimulated exosomes or their loaded hydrogels and the differences in terms of origination and function between exosomes and the other kinds of EVs (microvesicles and apoptotic bodies) and mesenchymal stem cell lysates which provided a fundamental understanding on the specific application of different EVs and the future development.

Overall, this Research Topic covers recent advances in designing of bioactive soft materials and their applications for drug delivery and tissue repair. The editors hope that the current Research Topic will contribute to the research and development in the field of soft materials for drug delivery and tissue repair, inspiring future exploration to broad the biomedical applications of bioactive soft materials.

